# The burden of dengue fever in travellers: a systematic literature review

**DOI:** 10.1016/j.nmni.2025.101631

**Published:** 2025-08-28

**Authors:** Martin P. Grobusch, Marta Díaz-Menéndez, Eduardo Bittencourt de Gomensoro, Caroline Mächler, Bojana Milovanović

**Affiliations:** a Centre for Tropical Medicine and Travel Medicine, Department of Infectious Diseases, Division of Internal Medicine, Amsterdam Public Health-Global Health, Amsterdam Infection & Immunity, Amsterdam University Medical Centre, Location University of Amsterdam, Amsterdam, Netherlands; b Institute of Tropical Medicine and DZIF Partner Site Tübingen, German Center for Infectious Disease Research, University of Tübingen, Tübingen, Germany; c Centre de Recherches Médicales en Lambaréné (CERMEL), Lambaréné, Gabon; d Masanga Medical Research Unit (MMRU), Masanga, Sierra Leone; e Institute of Infectious Diseases and Molecular Medicine (IDM), University of Cape Town, Cape Town, South Africa; f National Referral Centre for Imported Diseases and International Health, Hospital Universitario La Paz, IdiPAZ, CIBERINFEC, Madrid, Spain; g Takeda Pharmaceuticals International AG, Zurich, Switzerland

**Keywords:** Dengue, Travellers, Epidemiology, Healthcare resource use, Systematic literature review

## Abstract

**Background:**

Dengue is a mosquito-borne viral infection with growing global impact, including international travellers travelling to and from endemic regions. This systematic literature review aimed to assess the clinical and economic burden of dengue in travellers from non-endemic countries.

**Methods:**

This systematic review was conducted following the PRISMA guidelines to assess the incidence, prevalence, mortality, healthcare resource use, and costs of dengue fever in travellers between non-endemic and endemic regions. We systematically searched MEDLINE, Embase, Google Scholar, PubMed, and Epistemonikos. Due to heterogeneity, data were synthesised descriptively without quantitative analysis.

**Results:**

Seventy unique studies were included. Heterogeneity in design, inconsistent reporting, and lack of denominator data limited the ability to calculate incidence rates or compare risks across regions. Two prospective studies provided incidence rates, reaching 58.7 cases per 1000 person-months of travel. No studies reported prevalence data. With three cases, reported mortality was rare. No cost data were identified, but limited data on healthcare resource use were identified from seven publications.

**Conclusions:**

Findings highlight the challenges in quantifying individual risk and the need for improved surveillance, prospective data collection, and targeted prevention strategies. These results support improved travel health guidance and may inform vaccination strategies for travellers to endemic regions.

## 1 Introduction

Dengue fever, a mosquito-borne viral illness caused by four distinct serotypes of the dengue virus —DENV-1, DENV-2, DENV-3, and DENV-4—continues to present a substantial global public health challenge. Currently, dengue is considered endemic in most countries with sustained transmission in regions where mean annual temperatures exceed 18 °C [1]. Optimal conditions for viral replication and mosquito vector activity occurs at temperatures between 25 °C and 29 °C. The rainy season further exacerbates transmission by increasing standing water sites conducive to mosquito breeding [2]. The severity of the infection is dependent on many factors, but, at least in areas where dengue is endemic, secondary dengue infections, among others, is a risk factor to develop severe disease due to antibody-dependent enhancement (ADE) [3]. However, although ADE has historically been considered a key mechanism underlying severe dengue during secondary infections, recent evidence suggests that severe outcomes can also occur in primary infections, particularly among travellers from non-endemic regions. As such, the role of ADE in dengue pathogenesis may be more complex and less predictable than previously thought [4].

Like other arboviral infections such as Zika and chikungunya, dengue follows cyclical patterns characterized by periodic outbreaks and shifts in dominant serotypes, often influenced by both environmental and human behavioral factors [5]. Dengue has emerged as the leading cause of febrile illness among returning travellers from tropical regions, excluding Africa [6]. Furthermore, climate change [7] and growing urbanization [8] are expected to increase the burden of dengue in the future.

Currently, there are no specific antiviral treatments available for dengue, and the development of effective treatments remains a major challenge [9]. Consequently, prevention is prioritized, with mosquito bite avoidance strategies forming the cornerstone of current recommendations for travellers. Dengue vaccination has recently emerged as a preventive option to reduce the risk and severity of illness both in endemic areas as well as for the protection of travellers from non-endemic countries, with TAK-003 to be the only available EMA-approved vaccine by the end of 2025 following the discontinuation of Dengvaxia [[10], [11], [12], [13]]. Dengue poses a significant global burden, both clinically and economically. A global estimate placed the annual number of symptomatic dengue infections at nearly 60 million, resulting in approximately 13,500 deaths and incurring economic costs of US $9 billion [14]. These figures likely underestimate the true burden, and both the incidence and associated costs have continued to rise [15,16].

Despite growing recognition of dengue's impact, especially in mobile populations, existing studies on the disease burden in travellers are often anecdotal or limited in scope. To our knowledge, this review is the first to systematically review published data on the clinical and economic burden of dengue in this group without geographic limitations.

To address this gap, the objective of this systematic literature review (SLR) was to identify and synthesize published evidence on the clinical and economic burden of dengue in international travellers, travelling to and from endemic areas.

## 2 Methods

This SLR was conducted to identify and describe evidence on incidence, prevalence, mortality, healthcare resource use (HCRU), and costs associated with dengue fever (symptomatic, asymptomatic, confirmed or suspected) in travellers from non-endemic countries travelling to and from endemic areas. The study was registered on PROSPERO [17] (CRD42024539702) and developed in accordance with the PRISMA statement [18].

Searches were conducted in MEDLINE and Embase (Supplementary Tables 1–4), with supplementary searches in Google Scholar, PubMed, and Epistemonikos.

Study selection followed predefined population, intervention, comparator(s), outcomes, and study design (PICOS) criteria (Table 1), and all primary studies were eligible except case reports. Screening and selection were performed independently by two reviewers, with discrepancies resolved by a third.

**Table 1:** Eligibility criteria for study selection.

**Population**	• **Travellers from non-endemic areas travelling to and from endemic dengue fever areas**
**Intervention**	• No limitations
**Comparator**	• No limitations
**Outcomes**	• Incidence and prevalence of dengue fever^a^ in travellers from non-endemic areas coming to and from endemic dengue fever areas • Mortality associated with dengue fever^a^ in travellers from non-endemic areas coming to and from endemic dengue fever areas • Direct costs (e.g., drug/treatment costs, hospitalizations) and indirect costs (e.g., absenteeism, work productivity) associated with the treatment of dengue fever^a^ • Resource use (e.g., hospitalizations, outpatient visits) associated with the treatment of dengue fever^a^
**Study design**	• Any study design published in a peer-reviewed manuscript reporting outcomes of interest^b^
**Timeframe**	• 1 January 2009–30 April 2024
**Language**	• No limitations^c^

**Abbreviations:** PICOS, population, intervention, comparator, outcomes and study design.

a Symptomatic, asymptomatic, suspected, and confirmed cases of dengue fever (dengue 1–4 infection) were included.

b Although literature reviews and meta-analyses were excluded, we reviewed their bibliographies during the screening process to cross-check primary articles were retrieved from the literature searches. Any potentially relevant articles that had not been retrieved from the literature searches were hand searched.

c Non-English language articles were translated using software.

Only studies reporting travel between non-endemic and endemic countries were included. Endemicity was defined using the European Centre for Disease Prevention and Control's (ECDC) threshold of ≥10 cases per 100,000 population annually [19], aided by the most recent World Health Organization (WHO) geographic data [20]. Outcomes of interest included the number of cases, incidence, prevalence, disease severity, infecting serotype, primary or secondary infection, mortality, HCRU, and costs. Due to heterogeneity, data were synthesised descriptively without quantitative analysis.

## 3 Results

**Fig. 1:**
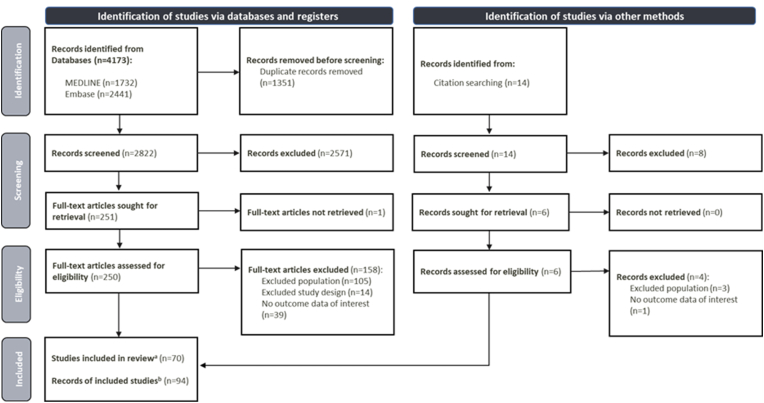
PRISMA flow chart [18] ^a^ Number of unique studies included in the review ^b^ Number of publications reporting on the 70 unique studies included in the review Abbreviations: PRISMA, Preferred Reporting Items for Systematic Reviews and Meta-Analyses.

Following deduplication, 2822 records were identified through electronic databases, and their titles/abstracts were screened (Fig. 1). A total of 251 full-text articles were assessed for eligibility. Of these, 92 publications met the inclusion criteria; 68 of the publications represented unique studies. A further two studies were identified through hand searching, resulting in a total of 70 studies included in the SLR. A complete list of included publications is provided in Supplementary Table 5, and excluded full-text articles, with reasons for exclusion, are detailed in Supplementary Table 6.

The included studies were heterogeneous in design. The majority were observational, cross-sectional in nature, and population-based, with data collected at a single time point or over a short duration. These studies permitted the identification of associations but not causal relationships [21]. Only two studies were identified as truly prospective [22,23]. Due to methodological variability, formal critical appraisal was not feasible; however, the overall risk of bias and confounding was considered high, limiting the strength of interpretation. Study sample sizes varied widely, ranging from eight participants [24] to over 125 million [25].

Participant characteristics from the included studies are summarized in Table 2. Demographic data such as age, sex, race, and comorbidities were inconsistently reported. Travel characteristics are detailed in Table 3. The median duration of travel was 19 days across two studies (n = 570) [23,26]. The main reason for travel was tourism, reported in five studies (n = 591) [22,23,[26], [27], [28]], followed by business or military service (n = 583) [26,29], and visiting friends and relatives (n = 247) [22,23,26,30]. Most studies reported only a single destination country.

**Table 2:** Characteristics of the participants in the included studies.

Patient demographics			Patients (n)	Publications (N)	References
**Age/sex (publications N=12)**					
Age (years)	Range of means	11.65–49.6	510	4	[29,30,33,52]
	Range of medians	35–57	1765	3	[23,26,35]
Male	n (%)	1855 (66.1)	2805	12	[23,26,27,29,30,33,35,37,38,[52], [53], [54]]
**Ethnicity (publications N=2)**					
Asian, Pacific Islander	n (%)	4 (0.8)	494	1	[29]
Black, Non-Hispanic	n (%)	6 (1.2)	494	1	[29]
Chinese	n (%)	1195 (100)	1195	1	[35]
White, Hispanic	n (%)	438 (88.7)	494	1	[29]
White, Non-Hispanic	n (%)	40 (8.1)	494	1	[29]
Other or unknown	n (%)	6 (1.2)	494	1	[29]
**Comorbidities (publications N=1)**					
Diabetes mellitus	n (%)	4 (0.3)	1195	1	[35]
Hypertension	n (%)	18 (1.5)	1195	1	[35]

**Table 3:** The nature of travel described in studies which reported it.

Travel and disease duration (publications N = 8)					
Factor	Data measure	Result	Participants (n)	Studies (N)	References
Duration of travel (days)	Range of means	38.0–50.2	21	2	[22,30]
	Range of medians	16–22	570	2	[23,26]
Time between travel and onset of symptoms (days)	Range	0–14	300	3	[27,39,55]
Disease duration (days)	Median	5	1195	1	[35]
Reason for travel (N = 8)					
Tourism	n (%)	541 (91.5)	591	5	[22,23,[26], [27], [28]]
Business/Military Service	n (%)	507 (87.0)	583	2	[26,29]
Visiting friends and relatives	n (%)	157 (63.6)	247	4	[22,23,26,30]
Education/Research	n (%)	17 (14.3)	119	3	[22,26,56]
Voluntary/Missionary work	n (%)	8 (7.4)	108	2	[22,26]
Migration	n (%)	6 (6.7)	89	1	[26]
Planned medical care	n (%)	2 (2.2)	89	1	[26]
No. countries visited during exposure period (N = 17)					
1 country	n (%)	3149 (99.3)	3171	17	[[22], [23], [24],^27^,^29^,^30^,^33^,^35^,^39^,^55^,[57], [58], [59], [60], [61], [62], [63], [64]]
2 countries	n (%)	4 (0.1)	3148	15	[[22], [23], [24],^27^,^29^,^30^,^33^,^35^,^39^,[58], [59], [60], [61], [62], [63]]
≥3 countries	n (%)	7 (0.2)	3148	15	[[22], [23], [24],^27^,^29^,^30^,^33^,^35^,^39^,[58], [59], [60], [61], [62], [63]]

Approximately half of the studies described dengue diagnoses as ‘confirmed’. Disease severity was reported in ten studies; however, inconsistent terminology precluded reliable classification. Additionally, while several studies reported dengue serotype, data were often incomplete, limiting interpretation. Serotype data were reported in 19 studies involving 2783 travellers, with DENV-1 being the most common (n = 1225; 44.0 %), followed by DENV-2 (n = 829; 29.8 %), DENV-3 (n = 474; 17.0 %), and DENV-4 (n = 255; 9.2 %). Information on infection history was limited. Three studies reported only primary infections [24,31,32], while one study (N = 8) identified two secondary and six primary infections [33].

**Fig. 2:**
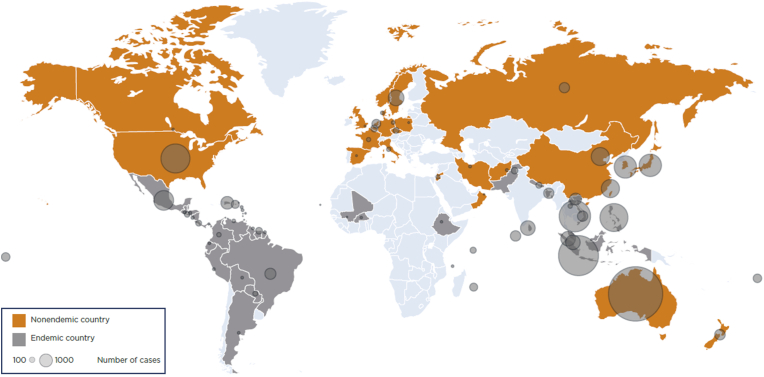
Events of dengue in travellers, by geographical region Key: The circles on the non-endemic countries of the map show the number of cases of dengue in travellers from these areas, while circles in endemic countries show where the infections were acquired. The size of the circles is proportional to the number of cases reported.

Nearly all the studies (68/70; 97.14 %) reported event data (occurrence of dengue), with 40/68 (58.82 %) providing definitive diagnostic information. The aggregated number of dengue cases is shown in Fig. 2. In total, 25 non-endemic countries reported imported dengue cases from 67 endemic countries or territories. Only two studies reported incidence rates: one from the Netherlands reported an incidence rate (IR) of 47.0 per 1000 person-months in travellers to Suriname [23], and another from the USA reported a seroconversion IR of 58.7 per 1000 person-months (95 % CI: 39.2 to 78.1) in travellers to and from multiple dengue-endemic endemic countries in the Caribbean, Latin America, Asia, and Africa [22].

**Fig. 3:**
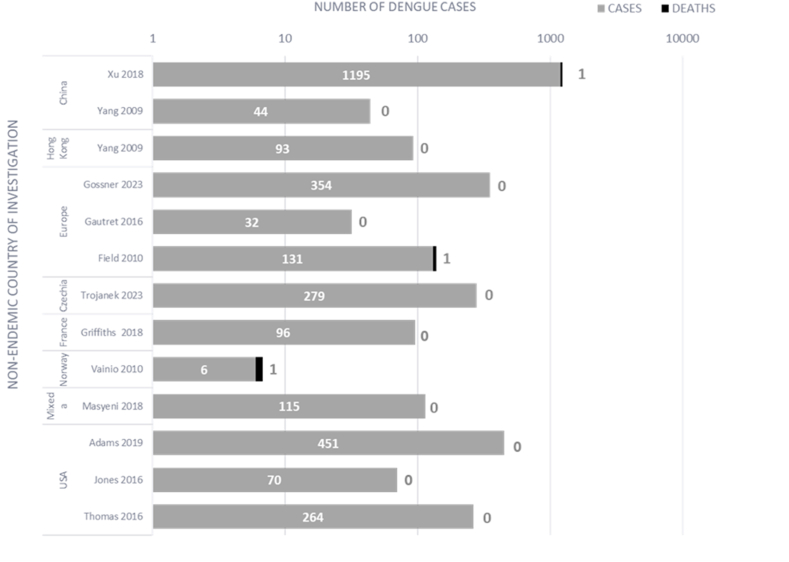
The number of deaths in travellers with dengue, in those studies which reported it ^a^ Germany, Russia, The Netherlands, Sweden, France, USA, Australia, Belgium, UK, Spain, Norway, Denmark, Japan.

The occurrence of dengue leading to mortality was reported in 12 studies from 26 endemic countries, with three of these studies reporting a single death in the reported cohort [24,34,35] (Fig. 3). The fatal cases were in patients who had acquired dengue from either Thailand (n = 2) [24,34] or Singapore (n = 1) [35]. Two patients died from dengue shock syndrome [24,34]. In the only study reporting infection history, the death was caused by a primary infection [24].

No data were identified on the costs of dengue management in travellers. However, limited data on HCRU were identified from seven publications. Hospitalisation rates among infected travellers ranged from 3.2 to 100 % [30,[36], [37], [38], [39]], with lengths of stay ranging from one to 27 days [30,35,[38], [39], [40]]. One study reported medical evacuation in 4/44 cases (2 %) [36], and another reported intensive care unit admission in 2/1995 cases (0.2 %) [35].

## 4 Discussion

The impact of dengue on international travellers has been recognised anecdotally for decades, with one of the earliest reported fatal travel-related cases documented in 1998 [41]. However, the full extent of the clinical and economic burden remains unclear. SLRs offer a robust method for synthesizing available evidence to address such knowledge gaps. Despite the growing importance of travel-related dengue, few SLRs exist in this field. One prior SLR by Gwee et al. (2021) focused on the global importation patterns of dengue but did not explore infection characteristics in detail [42]. In contrast, the present review aimed to provide a broader analysis, identifying studies not only on case importation but also stratifying infection risk by country, and examining infection severity, serotype distribution, and HCRU, with no restriction on reporting sites.

While most included studies reported imported dengue cases, the absence of denominator and travel duration data limited the ability to calculate IRs or make standardized comparisons. Hence, the number of dengue cases, as reported in Fig. 2, likely reflects the geographical focus and availability of existing studies rather than actual incidence patterns. This lack of denominator data is a known limitation in travel medicine, largely due to reliance on passive surveillance systems [43]. Several factors contribute to the difficulty in calculating accurate IRs in travellers: the absence of a comprehensive global database capturing traveller numbers, limited data on outbound and return travel patterns, inadequate reporting on travel duration and purpose, and the likelihood of under-reporting or misdiagnosis [43]. Currently, no international surveillance system exists that can provide the necessary data for precise IR estimates. Networks such as GeoSentinel are useful for identifying travel-related illnesses but lack information on the total travelling population, restricting their utility for epidemiological risk assessment [44].

In the absence of routine data sources providing reliable denominators, prospective studies are essential to accurately estimate the incidence of dengue in travellers. We identified two studies that captured these data robustly. Overbosch et al. (2023) assessed the risk of dengue, Zika, and chikungunya among Dutch travellers to Suriname (n = 481), using serological testing and travel diaries [23]. Their findings highlight a substantial infection risk for travellers, particularly tourists, reinforcing the importance of tailored pre-travel health advice and preventive measures in high-risk regions. Olivero et al. (2016) conducted a prospective study among 589 travellers from the Boston Area Travel Medicine Network, using paired serological samples and questionnaires [22]. Their findings underscore the significant risk of dengue across diverse endemic regions, even during short-term travel. The proportion of symptomatic seroconversions highlights the clinical burden and reinforces the need for targeted preventive strategies. From these two prospective studies, the available data offer the most robust indication of traveller risk to date, although generalisability remains limited and highlights the need for improved surveillance. Two additional studies provided crude IR estimates, but their methodological limitations reduce confidence in the findings. Valerio et al. (2021) [45] and Vinner et al. (2012) [46] relied on indirect methods and population-level denominators, which do not accurately reflect individual travel exposure.

In addition to the prospective studies included in this review, recent epidemiological analyses also provide evidence of the burden of dengue importation into non-endemic settings. Hedrich et al. (2025), in a systematic review of *Aedes*-borne infections in Europe, found that the overwhelming majority of reported cases (94 %) were travel-related, with over 45,000 dengue cases imported to Europe between 2000 and 2023. Twelve deaths were linked to dengue, underlining its potential severity despite its low case-fatality rate in travellers [47]. Similarly, Brem et al. (2024) documented the role of viraemic returned travellers in seeding local outbreaks of autochthonous dengue across southern Europe, highlighting how imported cases can amplify into sustained transmission in the presence of competent vectors [48]. More recently, Arulmukavarathan et al. (2024) reported record numbers of imported dengue in France in 2024, with over 4000 cases, more than half of which originated in Guadeloupe and Martinique. This surge in importations coincided with an unprecedented number of local transmission events, reinforcing the interconnectedness of travel-related dengue and local epidemiology [49]. Together, these studies illustrate the dual role of travellers as both a high-risk group for infection and as a key driver of onward transmission in Europe.

We also sought to understand the nature of dengue infections in affected individuals. DENV-1 appeared to be the most common serotype, followed by DENV-2, DENV-3, and DENV-4. However, these findings are subject to methodological limitations, and the infecting serotype is largely determined by the prevailing strain at the time and location of exposure, as most travellers lack immunity. We identified four studies that used serology to distinguish between primary and secondary infections. Of 246 individuals tested, only 2 (0.45 %) had evidence of secondary dengue. This result is expected, as most travellers have no prior exposure to dengue. Importantly, this has implications for vaccination, since TAK-003 is considered less effective in individuals without a previous infection, including travellers. While concerns regarding antibody-dependent enhancement and vaccination in naïve individuals from endemic regions are valid, these issues may be less applicable to travellers [3]. Our review indicates that symptomatic and severe dengue in this group most often occurs as a primary infection.

A traveller's risk of dengue exposure and severity is influenced by travel frequency, duration, and destination, although these factors were not consistently quantifiable. However, individual studies, such as Pollett et al. (2022), highlight high-risk groups like military personnel, who face increased exposure due to prolonged stays in endemic areas [29]. This reinforces the need for vaccination strategies tailored to travel purpose and individual risk, including consideration of ADE potential.

Dengue can cause serious illness in travellers, but inconsistent reporting limited assessment of severity. Although three deaths were identified in our review, drawing meaningful conclusions from this is not possible as data points were based on limited article numbers. True mortality is also underestimated, as case reports were excluded. A previous review by Huits and Schwartz (2021) identified nine traveller deaths from dengue in endemic areas [4]. Most (7/9); 78 %) cases were due to primary infection. The deceased were mainly relatively young women (8/9); 89 %) with a median age of 32 years (range 21–63). Nevertheless, the overall mortality rate of dengue in travellers appears to be rare, with a 15-year GeoSentinel survey reporting only one death [6]. This observation is consistent with findings from the 2023 GeoSentinel analysis by Huits et al., which also reported a single fatality among 86 cases of complicated dengue [50]. However, this figure may underestimate the true mortality burden, as fatal cases may not be detected in the country of origin if travellers die while still in the endemic country.

No cost data were found, and HCRU data were limited, but severe cases are likely to carry significant economic and societal burden, with the global economic impact projected at $10 billion annually in the coming decades [51]. Hospitalisation rates varied widely and may reflect national policies (e.g., hospitalising all dengue cases in some non-endemic settings), not only clinical severity. However, hospitalisation as a metric must be interpreted with caution since it may overestimate severity, especially in non-endemic countries where policies are conservative.

The strengths of this SLR include its systematic approach and broad scope, allowing for comprehensive coverage of relevant literature. However, as with previous reviews, interpretation was limited by heterogeneity in study design, diagnostic criteria, and case definitions, which made comparisons difficult. Underreporting, particularly of mild or asymptomatic cases, and selection bias likely led to underestimation of dengue burden in travellers. Additionally, variability in traveller characteristics, destinations, and timing further complicated comparisons, and the lack of denominator data prevented accurate incidence calculations.

Due to these limitations, we could not extract reliable quantitative data or compare risks between countries to inform vaccination strategies. Further secondary research is unlikely to close these gaps, and large-scale prospective studies are probably unrealistic due to the logistics and costs entailed, with questions over funding. Alternatively, hybrid approaches could be undertaken using mathematical modelling to combine case data (e.g., from the GeoSentinel survey) with travel volume statistics (e.g., flight data, visa entries) to offer a viable alternative for estimating individual traveller risk. Given the evolving nature of dengue epidemiology, accurately quantifying individual travel risk continues to be a challenge.

## 5 Conclusions

This SLR highlights the substantial yet under-characterized burden of dengue in international travellers. While the risk of infection is evident, gaps in surveillance data, inconsistent reporting, and lack of denominator information limit accurate risk quantification and cross-country comparisons. Despite the limitations of the available data, the findings have important public health and vaccination implications, particularly in informing risk communication, travel advice, and targeted prevention strategies for non-immune travellers. Most infections appear to be primary, with symptomatic and severe cases occurring even among short-term travellers. Although mortality remains rare, the clinical and potential economic impact is notable. These findings emphasize the need for improved data collection but confirm that research into this field is challenging and quantifying the risk of dengue at individual level is currently not practically achievable.

## CRediT authorship contribution statement

**Martin P. Grobusch:** Writing – review & editing. **Marta Díaz-Menéndez:** Writing – review & editing. **Eduardo Bittencourt de Gomensoro:** Writing – review & editing, Conceptualization. **Caroline Mächler:** Writing – review & editing. **Bojana Milovanović:** Writing – review & editing, Conceptualization.

## Author disclosures

MPG serves as Takeda Dengue Advisory board member (no personal payments received). MDM: Nothing to disclose. EBG, CM, and BM: Employees of Takeda and hold stock/stock options in Takeda.

## Funding

This work was supported by 10.13039/100016469Takeda Pharmaceuticals International AG.

## Declaration of competing interest

The authors declare the following financial interests/personal relationships which may be considered as potential competing interests:MPG serves as Takeda Dengue Advisory board member (no personal payments received). MDM: Nothing to disclose. EBG, CM, and BM: Employees of Takeda and hold stock/stock options in Takeda.
