# Supplementary material for: The burden of dengue fever in travellers: a systematic literature review
Source: New Microbes New Infect. 2025 Aug 28;67:101631. doi: 10.1016/j.nmni.2025.101631 (PMC12414826; doi:10.1016/j.nmni.2025.101631)
Supplement: Multimedia component 1 [file mmc1.docx]

**Supplementary materials**

**Table S1.** Incidence, prevalence, and mortality search strategy – MEDLINE

| **#** | **Searches** | **Results** |
| --- | --- | --- |
| **1** | exp Dengue/ | 16635 |
| **2** | dengue.mp. | 30478 |
| **3** | ((aden or bouguet or break-bone or breakbone or "brak bone" or dandy or solar or sun) adj2 fever*).mp. | 42 |
| **4** | or/1-3 | 30489 |
| **5** | Travel/ or Tourism/ or Travel Medicine/ or Travel-Related Illness/ or Military Deployment/ or Military Personnel/ or Missionaries/ or "Transients and Migrants"/ | 90186 |
| **6** | (travel* or emporiatric* or touris* militar* or soldier* or troop* or expat* or missionar* or migrant* or immigrant* or emigrant* or refugee*).ti,ab,kf. | 168834 |
| **7** | or/5-6 | 222570 |
| **8** | Epidemiology/ | 12595 |
| **9** | epidemiolog*.mp. | 2480732 |
| **10** | Incidence/ | 306229 |
| **11** | incidence.mp. | 1070725 |
| **12** | Prevalence/ | 351809 |
| **13** | prevalence.mp. | 928960 |
| **14** | Mortality/ | 49839 |
| **15** | mortality.mp. | 1456607 |
| **16** | Death/ | 20804 |
| **17** | death$.mp. | 1172045 |
| **18** | or/8-17 | 5134466 |
| **19** | 4 and 7 and 18 | 1210 |
| **20** | limit 19 to last 15 years | 908 |
| **Total** | | **908** |

MEDLINE: Searched on 30/04/24 via OvidSP interface. Database: Ovid MEDLINE(R) and Epub Ahead of Print, In-Process, In-Data-Review & Other Non-Indexed Citations, Daily and Versions <1946 to April 29, 2024>

**Table S2**. Incidence, prevalence, and mortality search strategy – Embase

| **#** | **Searches** | **Results** |
| --- | --- | --- |
| **1** | exp dengue/ | 29681 |
| **2** | dengue.mp. | 43643 |
| **3** | ((aden or bouguet or break-bone or breakbone or "brak bone" or dandy or solar or sun) adj2 fever*).mp. | 48 |
| **4** | or/1-3 | 43646 |
| **5** | travel/ or emporiatrics/ or travel related disease/ or tourism/ or travel/ or army/ or military deployment/ or migrant/ or military personnel/ | 111908 |
| **6** | (travel* or emporiatric* or touris* militar* or soldier* or troop* or expat* or missionar* or migrant* or immigrant* or emigrant* or refugee*).ti,ab,kf. | 206159 |
| **7** | or/5-6 | 251122 |
| **8** | epidemiology/ | 259929 |
| **9** | epidemiolog*.mp. | 1894406 |
| **10** | incidence/ | 606942 |
| **11** | incidence.mp. | 1582257 |
| **12** | prevalence/ | 999105 |
| **13** | prevalence.mp. | 1453182 |
| **14** | mortality/ | 928661 |
| **15** | mortality.mp. | 1996485 |
| **16** | death/ | 293295 |
| **17** | death$.mp. | 1819121 |
| **18** | or/8-17 | 6547286 |
| **19** | 4 and 7 and 18 | 1596 |
| **20** | limit 19 to conference abstract | 242 |
| **21** | 19 not 20 | 1354 |
| **22** | limit 21 to last 15 years | 947 |
| **Total** | | **947** |

Embase: Searched 30/04/24 via OvidSP interface. Database: Embase <1974 to 2024 April 29>

**Table S3.** HCRU and costs search strategy – MEDLINE

| **#** | **Searches** | **Results** |
| --- | --- | --- |
| **1** | exp Dengue/ | 16635 |
| **2** | dengue.mp. | 30478 |
| **3** | ((aden or bouguet or break-bone or breakbone or "brak bone" or dandy or solar or sun) adj2 fever*).mp. | 42 |
| **4** | or/1-3 | 30489 |
| **5** | exp Health Care Costs/ | 72881 |
| **6** | exp Employment/ | 102223 |
| **7** | exp Work/ | 71231 |
| **8** | "Cost of Illness"/ | 32233 |
| **9** | "Length of Stay"/ | 104336 |
| **10** | ((employment or employed or employee$ or unemployment or unemployed) adj3 (economic$ or cost or costs or costly or costing or price or prices or pricing)).ti,ab. | 3112 |
| **11** | (productivity adj3 (economic$ or cost or costs or costly or costing or price or prices or pricing)).ti,ab. | 4300 |
| **12** | ((long standing or longstanding or long term or longterm or permanent or employee$) adj2 (absence$ or absent$ or ill$ or sick$ or disab$)).ti,ab. | 13905 |
| **13** | llsi.ti,ab. | 16 |
| **14** | (cost$ adj2 (illness or disease$ or sickness$ or care or healthcare)).ti,ab. | 58459 |
| **15** | (burden$ adj2 (illness or disease$ or sickness$ or care or healthcare)).ti,ab. | 51572 |
| **16** | ((social or societ$ or work$ or employe$ or business$ or communit$ or famil$ or carer$ or caregiver$) adj3 (burden$ or consequenc$ or impact$ or problem$ or productivity or sickness or impairment$)).ti,ab. | 139414 |
| **17** | ((allowance or status or long-term or pension$ or benefit$) adj2 disab$).ti,ab. | 17492 |
| **18** | ((unable or inability or incapacit$ or incapab$) adj3 work).ti,ab. | **2271** |
| **19** | budget$ impact$.ti,ab. | 2404 |
| **20** | budget$ implicat$.ti,ab. | 87 |
| **21** | resource$ use$.ti,ab. | 13902 |
| **22** | resource$ utili$.ti,ab. | 16671 |
| **23** | resource$ usage.ti,ab. | 681 |
| **24** | (length adj2 stay$).ti,ab. | 84760 |
| **25** | (hospital$ adj2 stay$).ti,ab. | 121990 |
| **26** | (duration adj2 stay$).ti,ab. | 5029 |
| **27** | extended stay$.ti,ab. | 281 |
| **28** | prolonged stay$.ti,ab. | 1215 |
| **29** | ((hospitali?ation$ or hospitali?ed) adj3 (economic$ or cost or costs or costly or costing or price or prices or pricing)).ti,ab. | 11152 |
| **30** | economic consequenc$.ti,ab. | 4972 |
| **31** | or/5-30 | 737038 |
| **32** | 4 and 31 | 912 |
| **33** | limit 32 to last 15 years | 824 |
| **Total** | | **824** |

MEDLINE: Searched on 30/04/24 via OvidSP interface. Database: Ovid MEDLINE(R) and Epub Ahead of Print, In-Process, In-Data-Review & Other Non-Indexed Citations, Daily and Versions <1946 to April 29, 2024>

**Table S4**. HCRU and costs search strategy – Embase

| **#** | **Searches** | **Results** |
| --- | --- | --- |
| **1** | exp dengue/ | 29681 |
| **2** | dengue.mp. | 43643 |
| **3** | ((aden or bouguet or break-bone or breakbone or "brak bone" or dandy or solar or sun) adj2 fever*).mp. | 48 |
| **4** | or/1-3 | 43646 |
| **5** | exp "health care cost"/ | 351875 |
| **6** | exp employment/ | 132804 |
| **7** | exp work/ | 449874 |
| **8** | "cost of illness"/ | 21648 |
| **9** | "length of stay"/ | 287062 |
| **10** | ((employment or employed or employee$ or unemployment or unemployed) adj3 (economic$ or cost or costs or costly or costing or price or prices or pricing)).ti,ab. | 3888 |
| **11** | (productivity adj3 (economic$ or cost or costs or costly or costing or price or prices or pricing)).ti,ab. | 6031 |
| **12** | ((long standing or longstanding or long term or longterm or permanent or employee$) adj2 (absence$ or absent$ or ill$ or sick$ or disab$)).ti,ab. | 19330 |
| **13** | llsi.ti,ab. | 18 |
| **14** | (cost$ adj2 (illness or disease$ or sickness$ or care or healthcare)).ti,ab. | 90838 |
| **15** | (burden$ adj2 (illness or disease$ or sickness$ or care or healthcare)).ti,ab. | 78716 |
| **16** | ((social or societ$ or work$ or employe$ or business$ or communit$ or famil$ or carer$ or caregiver$) adj3 (burden$ or consequenc$ or impact$ or problem$ or productivity or sickness or impairment$)).ti,ab. | 183108 |
| **17** | ((allowance or status or long-term or pension$ or benefit$) adj2 disab$).ti,ab. | 28277 |
| **18** | ((unable or inability or incapacit$ or incapab$) adj3 work).ti,ab. | 3397 |
| **19** | budget$ impact$.ti,ab. | 6244 |
| **20** | budget$ implicat$.ti,ab. | 129 |
| **21** | resource$ use$.ti,ab. | 21109 |
| **22** | resource$ utili$.ti,ab. | 30291 |
| **23** | resource$ usage.ti,ab. | 944 |
| **24** | (length adj2 stay$).ti,ab. | 159486 |
| **25** | (hospital$ adj2 stay$).ti,ab. | 202975 |
| **26** | (duration adj2 stay$).ti,ab. | 7922 |
| **27** | extended stay$.ti,ab. | 437 |
| **28** | prolonged stay$.ti,ab. | 2022 |
| **29** | ((hospitali?ation$ or hospitali?ed) adj3 (economic$ or cost or costs or costly or costing or price or prices or pricing)).ti,ab. | 19812 |
| **30** | economic consequenc$.ti,ab. | 6315 |
| **31** | or/5-30 | 1604201 |
| **32** | 4 and 31 | 2127 |
| **33** | limit 32 to conference abstract | 439 |
| **34** | 32 not 33 | 1688 |
| **35** | limit 34 to last 15 years | 1494 |
| **Total** | | **1494** |

Embase: Searched 30/04/24 via OvidSP interface. Database: Embase <1974 to 2024 April 29>

**Table S5**. Included full-text articles

| **Bibliography** |
| --- |
| Adams LE, Martin SW, Lindsey NP, Lehman JA, Rivera A, Kolsin J, et al. Epidemiology of Dengue, Chikungunya, and Zika Virus Disease in U.S. States and Territories, 2017. Am J Trop Med Hyg. 2019;101(4):884-90.  Alang N, Glavis-Bloom J, Alexander-Scott N, Mermel LA, Mileno MD. Surveillance of Travel-Related Mosquito-borne Illness in Rhode Island. R I Med. 2016;99(7):22-3.  Al Awaidy ST, Al Obeidani I, Bawikar S, Al Mahrouqi S, Al Busaidy SS, Al Baqlani S, et al. Dengue epidemiological trend in Oman: a 13-year national surveillance and strategic proposition of imported cases. Trop Doct. 2014;44(4):190-5.  Ammar SE, McLntyre M, Baker MG, Hales S. Imported arboviral infections in New Zealand, 2001 to 2017: A risk factor for local transmission. Travel Med Infect Dis. 2021;41:102047.  Angelo KM, Haulman NJ, Terry AC, Leung DT, Chen LH, Barnett ED, et al. Illness among US resident student travellers after return to the USA: a GeoSentinel analysis, 2007-17. J Travel Med. 2018;25(1):01.  Avni C, Stienlauf S, Meltzer E, Sidi Y, Schwartz E, Leshem E. Region-Specific, Life-Threatening Diseases among International Travelers from Israel, 2004-2015. Emerg Infect Dis. 2018;24(4):790-3.  Boggild AK, Geduld J, Libman M, Yansouni CP, McCarthy AE, Hajek J, et al. Illness in Canadian travellers and migrants from Brazil: CanTravNet surveillance data, 2013-2016. Can Commun Dis Rep. 2016;42(8):153-7.  Burdino E, Milia MG, Sergi G, Gregori G, Allice T, Cazzato ML, et al. Diagnosis of dengue fever in North West Italy in travelers from endemic areas: a retrospective study. J Clin Virol. 2011;51(4):259-63.  Calleri G, Torta I, Gobbi F, Angheben A, Lipani F, Lucchini A, et al. [Imported dengue in two tertiary Italian hospitals: Use of rapid diagnostic tests]. Bull Soc Pathol Exot. 2017;110(1):13-9.  Chinikar S, Ghiasi SM, Shah-Hosseini N, Mostafavi E, Moradi M, Khakifirouz S, et al. Preliminary study of dengue virus infection in Iran. Travel Med Infect Dis. 2013;11(3):166-9.  Cho KH, Park SY, Lee WC, Lee MJ, Lee JB. International Travel and Exotic Dengue Fever in South Korea from 2006 to 2015. Jpn J Infect Dis. 2018;71(5):378-81.  Chong CH, McCaskill ME, Britton PN. Pediatric travelers presenting to an Australian emergency department (2014-2015): A retrospective, cross-sectional analysis. Travel Med Infect Dis. 2019;31:101345.  Cleton N, Reusken C, Murk JL, de Jong M, Reimerink J, van der Eijk A, et al. Using routine diagnostic data as a method of surveillance of arboviral infection in travellers: a comparative analysis with a focus on dengue. Travel Med Infect Dis. 2014;12(2):159-66.  Ding Z, Wu C, Wu H, Lu Q, Lin J. The Epidemiology of Imported Acute Infectious Diseases in Zhejiang Province, China, 2011-2016: Analysis of Surveillance Data. Am J Trop Med Hyg. 2018;98(3):913-9.  Ernst T, McCarthy S, Chidlow G, Luang-Suarkia D, Holmes EC, Smith DW, et al. Emergence of a new lineage of dengue virus type 2 identified in travelers entering Western Australia from Indonesia, 2010-2012. PLoS Negl Trop Dis. 2015;9(1):e0003442.  Feng X, Sun W, Birkhead GS, Wang X, Guo Z, Lu J. The surveillance of four mosquito-borne diseases in international travelers arriving at Guangzhou Baiyun International Airport, China, 2016-2017. Travel Med Infect Dis. 2019;32:101513.  Ferguson RW, Henderson SJ, Lee EA, Jung P. Dengue in Peace Corps Volunteers, 2000-14. J Travel Med. 2016;23(3).  Field V, Gautret P, Schlagenhauf P, Burchard GD, Caumes E, Jensenius M, et al. Travel and migration associated infectious diseases morbidity in Europe, 2008. BMC Infect Dis. 2010;10:330.  Fitzsimmons GJ, Wright P, Johansen CA, Whelan PI. Arboviral diseases and malaria in Australia, 2008-09: annual report of the National Arbovirus and Malaria Advisory Committee. Commun Dis Intell Q Rep. 2010;34(3):225-40.  Fukusumi M, Arashiro T, Arima Y, Matsui T, Shimada T, Kinoshita H, et al. Dengue Sentinel Traveler Surveillance: Monthly and Yearly Notification Trends among Japanese Travelers, 2006-2014. PLoS Negl Trop Dis. 2016;10(8):e0004924.  Gautret P, Mockenhaupt F, Grobusch MP, Rothe C, von Sonnenburg F, van Genderen PJ, et al. Arboviral and other illnesses in travellers returning from Brazil, June 2013 to May 2016: implications for the 2016 Olympic and Paralympic Games. Euro Surveill. 2016;21(27):07.  Gobbi F, Barzon L, Capelli G, Angheben A, Pacenti M, Napoletano G, et al. Surveillance for West Nile, dengue, and chikungunya virus infections, Veneto Region, Italy, 2010. Emerg Infect Dis. 2012;18(4):671-3.  Gossner CM, Hallmaier-Wacker L, Briet O, Haussig JM, de Valk H, Wijermans A, et al. Arthropod-borne diseases among travellers arriving in Europe from Africa, 2015 to 2019. Euro Surveill. 2023;28(7):02.  Gossner CM, Fournet N, Frank C, Fernandez-Martinez B, Del Manso M, Gomes Dias J, et al. Dengue virus infections among European travellers, 2015 to 2019. Euro Surveill. 2022;27(2):01.  Griffiths KM, Savini H, Brouqui P, Simon F, Parola P, Gautret P. Surveillance of travel-associated diseases at two referral centres in Marseille, France: a 12-year survey. J Travel Med. 2018;25(1):01.  Gulley CT, Murphy DE, Poe SA, Petersen K. A descriptive analysis of dengue in Peace Corps Volunteers, 2000-2019. Travel Med Infect Dis. 2021;43:102125.  Heddini A, Janzon R, Linde A. Increased number of dengue cases in Swedish travellers to Thailand. Euro Surveill. 2009;14(5):05.  Hirata K, Ogawa T, Fujikura H, Ogawa Y, Hirai N, Nakagawa-Onishi T, et al. Characteristics of health problems in returned overseas travelers at a tertiary teaching hospital in a suburban area in Japan. J Infect Chemother. 2018;24(8):682-5.  Huang JH, Su CL, Yang CF, Liao TL, Hsu TC, Chang SF, et al. Molecular characterization and phylogenetic analysis of dengue viruses imported into Taiwan during 2008-2010. Am J Trop Med Hyg. 2012;87(2):349-58.  Huang T, Zhou L, Shen T, Liu HH, He JB, Zheng ED, et al. [Epidemiological characteristics of imported acute infectious diseases in the border areas of Yunnan province, 2008-2017]. Chung Hua Liu Hsing Ping Hsueh Tsa Chih. 2019;40(4):412-6.  Huang X, Yakob L, Devine G, Frentiu FD, Fu SY, Hu W. Dynamic spatiotemporal trends of imported dengue fever in Australia. Sci. 2016;6:30360.  Jeong YE, Lee WC, Cho JE, Han MG, Lee WJ. Comparison of the Epidemiological Aspects of Imported Dengue Cases between Korea and Japan, 2006-2010. Osong Public Health Res Perspect. 2016;7(1):71-4.  Jones JM, Lopez B, Adams L, Galvez FJ, Nunez AS, Santillan NA, et al. Binational Dengue Outbreak Along the United States-Mexico Border - Yuma County, Arizona, and Sonora, Mexico, 2014. MMWR Morb Mortal Wkly Rep. 2016;65(19):495-9.  Kariyawasam R, Lau R, Eshaghi A, Patel SN, Sider D, Gubbay JB, et al. Spectrum of Viral Pathogens in Blood of Malaria-Free Ill Travelers Returning to Canada. Emerg Infect Dis. 2016;22(5):854-61.  Knope K, Giele C. Increasing notifications of dengue in Australia related to overseas travel, 1991 to 2012. Commun Dis Intell Q Rep. 2013;37(1):E55-9.  Knope KE, Muller M, Kurucz N, Doggett SL, Feldman R, Johansen CA, et al. Arboviral diseases and malaria in Australia, 2013-14: Annual report of the National Arbovirus and Malaria Advisory Committee. Commun Dis Intell Q Rep. 2016;40(3):E400-E36.  Kolsch Y, Phiri BSJ, Kupper T. Tropical infections as occupational diseases among young volunteers in social projects. Int J Hyg Environ Health. 2023;250:114164.  Kuna A, Bykowska M, Kulawiak N, Biernat B, Szostakowska B, Nahorski WL, Pawlowski W, Chomicz L. Clinico-laboratory profile of dengue patients returning from tropical areas to Poland during 2010-15. J Vector Borne Dis. 2016 Jul-Sep;53(3):234-9.  Kutsuna S, Hayakawa K, Kato Y, Fujiya Y, Mawatari M, Takeshita N, et al. Comparison of clinical characteristics and laboratory findings of malaria, dengue, and enteric fever in returning travelers: 8-year experience at a referral center in Tokyo, Japan. J Infect Chemother. 2015;21(4):272-6.  Lachish T, Lustig Y, Leshem E, Katz-Likvornik S, Biber A, Nadir E, et al. High incidence of dengue in Israel travelers to Kathmandu, Nepal, in 2019. J Travel Med. 2020;27(1):03.  Liu WH, Shi C, Lu Y, Luo L, Ou CQ. Epidemiological characteristics of imported acute infectious diseases in Guangzhou, China, 2005-2019. PLoS Negl Trop Dis. 2022 Dec 6;16(12):e0010940.  Luce R, Rivera A, Mohammed H, Tomashek KM, Lehman JA. Travel-associated Dengue surveillance - United States, 2006-2008. MMWR Morb Mortal Wkly Rep. 2010;59(23):715-9.  Lustig Y, Wolf D, Halutz O, Schwartz E. An outbreak of dengue virus (DENV) type 2 Cosmopolitan genotype in Israeli travellers returning from the Seychelles, April 2017. Euro Surveill. 2017;22(26):29.  Makela HMM, Cristea V, Sane JA. Lack of perception regarding risk of dengue and day-active mosquitoes in Finnish travellers. Infect Dis (Lond). 2020;52(9):651-8.  Masyeni S, Yohan B, Somia IKA, Myint KSA, Sasmono RT. Dengue infection in international travellers visiting Bali, Indonesia. J Travel Med. 2018;25(1):01.  Meltzer E, Avrami S, Lustig Y, Schwartz E. Incidence of dengue fever in Israeli travelers 2008-2019. Travel Med Infect Dis. 2022;48:102330.  Miki S, Lee WC, Lee MJ. A Comparative Study of the Trends of Imported Dengue Cases in Korea and Japan 2011 - 2015. J Clin Med Res. 2017;9(7):650-3.  Millman AJ, Esposito DH, Biggs HM, Decenteceo M, Klevos A, Hunsperger E, et al. Chikungunya and Dengue Virus Infections Among United States Community Service Volunteers Returning from the Dominican Republic, 2014. Am J Trop Med Hyg. 2016;94(6):1336-41.  Mizuno Y, Kato Y, Kano S, Takasaki T. Imported malaria and dengue fever in returned travelers in Japan from 2005 to 2010. Travel Med Infect Dis. 2012;10(2):86-91.  Mohammed HP, Ramos MM, Rivera A, Johansson M, Munoz-Jordan JL, Sun W, et al. Travel-associated dengue infections in the United States, 1996 to 2005. J Travel Med. 2010;17(1):8-14.  Nakamura N, Arima Y, Shimada T, Matsui T, Tada Y, Okabe N. Incidence of dengue virus infection among Japanese travellers, 2006 to 2010. Western pac. 2012;3(2):39-45.  Navero-Castillejos J, Benitez R, Torner N, Munoz J, Camprubi-Ferrer D, Peiro-Mestres A, et al. Molecular Characterization of Imported and Autochthonous Dengue in Northeastern Spain. Viruses. 2021;13(10):23.  Neumayr A, Munoz J, Schunk M, Bottieau E, Cramer J, Calleri G, et al. Sentinel surveillance of imported dengue via travellers to Europe 2012 to 2014: TropNet data from the DengueTools Research Initiative. Euro Surveill. 2017;22(1):05.  O'Donnell FL, Fan M, Stahlman S. Surveillance for Vector-borne Diseases Among Active and Reserve Component Service Members, U.S. Armed Forces, 2016-2020. Msmr. 2021;28(2):11-5.  Olivero RM, Hamer DH, MacLeod WB, et al. Dengue virus seroconversion in travelers to dengue-endemic areas. The American Journal of Tropical Medicine and Hygiene 2016; 95(5): 1130  Olszynska-Krowicka M, Switaj K. [Dengue in patients hospitalized in the hospital ward of zoonoses and tropical diseases in Warsaw in years 2002-2011]. Przegl Epidemiol. 2011;65(4):571-5.  Overbosch FW, Schinkel J, Matser A, Koen G, Prange I, Prins M, et al. Dengue, chikungunya and Zika virus infections among Dutch travellers to Suriname: a prospective study during the introduction of chikungunya and Zika virus, 2014 to 2017. Euro Surveill. 2023;28(2):01.  Pagani G, Zanchetta N, Galimberti L, Oreni L, Passerini S, Giacomelli A, et al. Imported dengue fever: a 16-years retrospective analysis in Milan (Italy) and a brief review of the European literature. Infez Med. 2020;28(2):243-52.  Pan CY, Liu WL, Su MP, Chang TP, Ho HP, Shu PY, et al. Epidemiological analysis of the Kaohsiung city strategy for dengue fever quarantine and epidemic prevention. BMC Infect Dis. 2020;20(1):347.  Park SH, Lee MJ, Baek JH, Lee WC. Epidemiological aspects of exotic malaria and dengue fever in travelers in Korea. J Clin Med Res. 2011;3(3):139-42.  Pierro A, Varani S, Rossini G, Gaibani P, Cavrini F, Finarelli AC, et al. Imported cases of dengue virus infection: Emilia-Romagna, Italy, 2010. Clin Microbiol Infect. 2011;17(9):1349-52.  Pollett S, Kuklis CH, Barvir DA, Jarman RG, Romaine RM, Forshey BM, et al. The seroepidemiology of dengue in a US military population based in Puerto Rico during the early phase of the Zika pandemic. PLoS Negl Trop Dis. 2022;16(1):e0009986.  Rivera A, Adams LE, Sharp TM, Lehman JA, Waterman SH, Paz-Bailey G. Travel-Associated and Locally Acquired Dengue Cases - United States, 2010-2017. MMWR Morb Mortal Wkly Rep. 2020;69(6):149-54.  Rowe SL, Thevarajan I, Richards J, Gibney K, Simmons CP. The Rise of Imported Dengue Infections in Victoria, Australia, 2010-2016. Trop. 2018;3(1):21.  Rocklov J, Lohr W, Hjertqvist M, Wilder-Smith A. Attack rates of dengue fever in Swedish travellers. Scand J Infect Dis. 2014;46(6):412-7.  Sahak MN. Dengue fever as an emerging disease in Afghanistan: Epidemiology of the first reported cases. Int J Infect Dis. 2020;99:23-7.  Sayfullin MA, Zvereva NN, Karan LS, Grigoreva YE, Akinshina YA, Larichev VF, et al. [Characteristics of imported cases of Dengue fever and hemorrhagic Dengue fever in 2009-2019]. Vopr Virusol. 2022;67(4):322-30.  Shi L, Fu S, Wang L, Li X, Gu D, Liu C, et al. Surveillance of mosquito-borne infectious diseases in febrile travelers entering China via Shenzhen ports, China, 2013. Travel Med Infect Dis. 2016;14(2):123-30.  Shu PY, Su CL, Liao TL, Yang CF, Chang SF, Lin CC, et al. Molecular characterization of dengue viruses imported into Taiwan during 2003-2007: geographic distribution and genotype shift. Am J Trop Med Hyg. 2009;80(6):1039-46.  Sohail A, McGuinness SL, Lightowler R, Leder K, Jomon B, Bain CA, et al. Spectrum of illness among returned Australian travellers from Bali, Indonesia: a 5-year retrospective observational study. Intern Med J. 2019;49(1):34-40.  Sohail A, Anders KL, McGuinness SL, Leder K. The epidemiology of imported and locally acquired dengue in Australia, 2012-2022. J Travel Med. 2024;31(2):01  Su CP, Wang YY, Ku KC, Fang CT. Clinical and epidemiological characteristics of imported dengue fever among inbound passengers: Infrared thermometer-based active surveillance at an international airport. PLoS ONE. 2019;14(12):e0225840.  Tai AY, McGuinness SL, Robosa R, Turner D, Huang GK, Leder K, et al. Management of dengue in Australian travellers: a retrospective multicentre analysis. Med J Aust. 2017;206(7):295-300.  Tavakolipoor P, Schmidt-Chanasit J, Burchard GD, Jordan S. Clinical features and laboratory findings of dengue fever in German travellers: A single-centre, retrospective analysis. Travel Med Infect Dis. 2016;14(1):39-44.  Taylor-Salmon E, Hill V, Paul LM, Koch RT, Breban MI, Chaguza C, et al. Travel surveillance uncovers dengue virus dynamics and introductions in the Caribbean. Nat Commun. 2024;15(1):3508.  Thomas DL, Santiago GA, Abeyta R, Hinojosa S, Torres-Velasquez B, Adam JK, et al. Reemergence of Dengue in Southern Texas, 2013. Emerg Infect Dis. 2016;22(6):1002-7.  Trojanek M, Maixner J, Sojkova N, Kyncl J, Rohacova H, Maresova V, et al. Dengue fever in Czech travellers: A 10-year retrospective study in a tertiary care centre. Travel Med Infect Dis. 2016;14(1):32-8.  Trojanek M, Grebenyuk V, Mandakova Z, Sojkova N, Zelena H, Rohacova H, et al. Epidemiology of dengue, chikungunya and Zika virus infections in travellers: A 16-year retrospective descriptive study at a tertiary care centre in Prague, Czech Republic. PLoS ONE. 2023;18(2):e0281612.  Tuiskunen A, Hjertqvist M, Vene S, Lundkvist A. Dengue fever in returned Swedish travelers from Thailand. Infect Ecol Epidemiol. 2011;1.  Vainio K, Noraas S, Holmberg M, Fremstad H, Wahlstrom M, Anestad G, et al. Fatal and mild primary dengue virus infections imported to Norway from Africa and south-east Asia, 2008-2010. Euro Surveill. 2010;15(38):23.  Valerio L, Roure Diez S, Benitez R, Fernandez-Rivas G, Rivaya B, Exposito C, et al. [Intensified epidemiological surveillance of arbovirosis: First case of native dengue fever in Catalonia (Spain), Northern Metropolitan Area of Barcelona, 2018-2019]. Aten Primaria. 2021;53(1):73-80.  Vasquez V, Haddad E, Perignon A, Jaureguiberry S, Brichler S, Leparc-Goffart I, et al. Dengue, chikungunya, and Zika virus infections imported to Paris between 2009 and 2016: Characteristics and correlation with outbreaks in the French overseas territories of Guadeloupe and Martinique. Int J Infect Dis. 2018;72:34-9.  Verschueren J, Cnops L, van Esbroeck M. Twelve years of dengue surveillance in Belgian travellers and significant increases in the number of cases in 2010 and 2013. Clin Microbiol Infect. 2015;21(9):867-72.  Viennet E, Ritchie SA, Faddy HM, Williams CR, Harley D. Epidemiology of dengue in a high-income country: a case study in Queensland, Australia. Parasit Vectors. 2014;7:379.  Vinner L, Domingo C, Ostby AC, Rosenberg K, Fomsgaard A. Cases of travel-acquired dengue fever in Denmark 2001-2009. Clin Microbiol Infect. 2012;18(2):171-6.  Warrilow D, Northill JA, Pyke AT. Sources of dengue viruses imported into Queensland, australia, 2002-2010. Emerg Infect Dis. 2012;18(11):1850-7.  Wang YY, Tsay PK. Risk Factors Associated with Passengers with Imported Dengue Fever at International Airports in Taiwan. Int J Environ Res Public Health. 2022;19(17):05.  Wong JM, Rivera A, Volkman HR, Torres-Velasquez B, Rodriguez DM, Paz-Bailey G, et al. Travel-Associated Dengue Cases - United States, 2010-2021. Am J Transplant. 2023;23(9):1455-61.  Wright P, Fitzsimmons GJ, Johansen CA, Whelan PI. Arboviral diseases and malaria in Australia, 2009-10: annual report of the National Arbovirus and Malaria Advisory Committee. Commun Dis Intell Q Rep. 2012;36(1):70-81.  Wu Y, Liu MY, Wang JL, Zhang HY, Sun Y, Yuan Y, et al. Epidemiology of imported infectious diseases, China, 2014-18. J Travel Med. 2020;27(8):23.  Xu C, Pang J, Hsu JP, Leo YS, Lye DCB. Differences in clinical features and dengue severity between local and migrant Chinese with dengue infection in Singapore. PLoS ONE. 2018;13(8):e0201441.  Yang F, Ma SQ, He JF, Mai ZJ, Liang WJ, Cai MX, et al. [Epidemiological analysis of imported cases of dengue fever in Guangdong province and Hong Kong during 2004-2006 in China]. Chung Hua Liu Hsing Ping Hsueh Tsa Chih. 2009;30(1):42-4.  Yuan B, Nishiura H. Estimating the actual importation risk of dengue virus infection among Japanese travelers. PLoS ONE. 2018;13(6):e0198734.  Zvereva NN, Saifullin MA, Sayfullin RF, Erovichenkov AA, Bazarova MV, Pshenichnaya NY. Epidemiological and etiological features of travel-related febrile illnesses in hospitalized Russian children and adults: A single-centre, retrospective analysis in Moscow. Travel Med Infect Dis. 2020;34:101447. |

**Table S6**. Excluded full-text articles

| **Identification of studies via databases and registers** |
| --- |
| **Reason for exclusion: excluded population (n=105)** |
| Al-Abri SS, Abdel-Hady DM, Al Mahrooqi SS, Al-Kindi HS, Al-Jardani AK, Al-Abaidani IS. Epidemiology of travel-associated infections in Oman 1999-2013: A retrospective analysis. Travel Med Infect Dis. 2015;13(5):388-93.  Ali A, Rehman HU, Nisar M, Rafique S, Ali S, Hussain A, et al. Seroepidemiology of dengue fever in Khyber Pakhtunkhawa, Pakistan. Int J Infect Dis. 2013;17(7):e518-23.  Allwinn R. Significant increase in travel-associated dengue fever in Germany. Med Microbiol Immunol (Berl). 2011;200(3):155-9.  Askling HH, Lesko B, Vene S, Berndtson A, Bjorkman P, Blackberg J, et al. Serologic analysis of returned travelers with fever, Sweden. Emerg Infect Dis. 2009;15(11):1805-8.  Aubry M, Mapotoeke M, Teissier A, Paoaafaite T, Dumas-Chastang E, Giard M, et al. Dengue virus serotype 2 (DENV-2) outbreak, French Polynesia, 2019. Euro Surveill. 2019;24(29).  Baly A, Toledo ME, Rodriguez K, Benitez JR, Rodriguez M, Boelaert M, Vanlerberghe V, Van der Stuyft P. Costs of dengue prevention and incremental cost of dengue outbreak control in Guantanamo, Cuba. Trop Med Int Health. 2012 Jan;17(1):123-32.  Berberian G, Perez G, Mangano A, Borgnia D, Buchovsky A, Costa M, et al. Dengue beyond the tropics: a time-series study comprising 2015-2016 versus 2019-2020 at a children's hospital in the City of Buenos Aires. Arch Argent Pediatr. 2022;120(6):384-90.  Blaizot R, Ouattara E, Receveur MC, Mechain M, Pistone T, Malvy D, et al. Dermatoses in international travellers seen at Bordeaux teaching hospital travel clinic, 2015-2018: a GeoSentinel-based study. Clin Exp Dermatol. 2020;45(5):580-3.  Boggild AK, Geduld J, Libman M, Ward BJ, McCarthy AE, Doyle PW, et al. Travel-acquired infections and illnesses in Canadians: surveillance report from CanTravNet surveillance data, 2009-2011. Open Med. 2014;8(1):e20-32.  Boggild AK, Esposito DH, Kozarsky PE, Ansdell V, Beeching NJ, Campion D, et al. Differential diagnosis of illness in travelers arriving from Sierra Leone, Liberia, or Guinea: a cross-sectional study from the GeoSentinel Surveillance Network. Ann Intern Med. 2015;162(11):757-64.  Boggild AK, Geduld J, Libman M, Yansouni CP, McCarthy AE, Hajek J, et al. Spectrum of illness in migrants to Canada: Sentinel surveillance through CanTravNet. J Travel Med. 2019;26(2).  Bright A, Glynn-Robinson AJ, Kane S, Wright R, Saul N. The effect of COVID-19 public health measures on nationally notifiable diseases in Australia: preliminary analysis. Commun Dis Intell (2018). 2020;44:05.  Bykowska-Tumasz M, Wysocki O, Sikorska K. Epidemiological and clinical analysis of polish short-term and long-term travelers returning from tropical countries. Travel Med Infect Dis. 2023;55:102642.  Camprubi-Ferrer D, Cobuccio L, Van Den Broucke S, Genton B, Bottieau E, d'Acremont V, et al. Causes of fever in returning travelers: a European multicenter prospective cohort study. J Travel Med. 2022;29(2):21.  Camprubí-Ferrer D, Ramponi F, Balerdi-Sarasola L, Godoy A, Sicuri E, Muñoz J. Rapid diagnostic tests for dengue would reduce hospitalizations, healthcare costs and antibiotic prescriptions in Spain: A cost-effectiveness analysis. Enferm Infecc Microbiol Clin (Engl Ed). 2024 Jan;42(1):30-33.  Centers for Disease Control and Prevention (CDC). Dengue virus infections among travelers returning from Haiti--Georgia and Nebraska, October 2010. MMWR Morb Mortal Wkly Rep. 2011 Jul 15;60(27):914-7  Chaparro PE, de la Hoz F, Lozano Becerra JC, Repetto SA, Alba Soto CD. Internal travel and risk of dengue transmission in Colombia. Rev Panam Salud Publica. 2014;36(3):197-200.  Chatterjee SS, Sharma A, Choudhury S, Chumber SK, Bage R, Parkhe N, et al. Dengue fever in a south Asian metropolis: a report on 219 cases. Iran. 2017;9(3):174-85.  Choe YJ, Choe SA, Cho SI. Importation of travel-related infectious diseases is increasing in South Korea: An analysis of salmonellosis, shigellosis, malaria, and dengue surveillance data. Travel Med Infect Dis. 2017;19:22-7.  Cotter CJ, Tufa AJ, Johnson S, Matai'a M, Sciulli R, Ryff KR, et al. Outbreak of Dengue Virus Type 2 - American Samoa, November 2016-October 2018. MMWR Morb Mortal Wkly Rep. 2018;67(47):1319-22.  Dahl V, Wallensten A. Self-reported infections during international travel and notifiable infections among returning international travellers, Sweden, 2009-2013. PLoS ONE. 2017;12(7):e0181625.  Dam Larsen F, Jespersen S, Wejse C, Petersen E, Larsen CS. One-sixth of inpatients in a Danish infectious disease ward have imported diseases: A cross-sectional analysis. Travel Medicine and Infectious Disease. 2017;20:43-8.  Dammermann W, Haller IE, Singethan K, Vinnemeier CD, Hentschel F. Asymptomatic arbovirus and campylobacter infections in German travelers to Asia. Arch Virol. 2023;168(10):254.  D'Ortenzio E, Balleydier E, Baville M, Filleul L, Renault P. [Dengue fever in the Reunion Island and in South Western islands of the Indian Ocean]. Med Mal Infect. 2011;41(9):475-9.  Esposito DH, Han PV, Kozarsky PE, Walker PF, Gkrania-Klotsas E, Barnett ED, et al. Characteristics and spectrum of disease among ill returned travelers from pre- and post-earthquake Haiti: The GeoSentinel experience. Am J Trop Med Hyg. 2012;86(1):23-8.  Fang LQ, Sun Y, Zhao GP, Liu LJ, Jiang ZJ, Fan ZW, et al. Travel-related infections in mainland China, 2014-16: an active surveillance study. Lancet Public Health. 2018;3(8):e385-e94.  Farag EA, Jaffrey S, Daraan F, Al-Shamali M, Khan FY, Coyle PV, et al. Dengue Epidemiology in Qatar from 2013-2021: A Retrospective Study. Trop. 2022;7(11):25.  Frank C, Lachmann R, Wilking H, Stark K. Increase in dengue fever in travellers returning from Egypt, Germany 2023. Euro Surveill. 2024;29(5).  Gautret P, Schlagenhauf P, Gaudart J, Castelli F, Brouqui P, von Sonnenburg F, et al. Multicenter EuroTravNet/GeoSentinel study of travel-related infectious diseases in Europe. Emerg Infect Dis. 2009;15(11):1783-90.  Gautret P, Gaudart J, Leder K, Schwartz E, Castelli F, Lim PL, et al. Travel-associated illness in older adults (>60 y). J Travel Med. 2012;19(3):169-77.  Goljan J, Myjak P, Nahorski W, Kubica-Biernat B, Felczak-Korzybska I, Kowalczyk D, et al. Dengue antibodies in Polish travellers returning from the tropics. Evaluation of serological tests. Int Marit Health. 2010;61(1):36-40.  Grobusch MP, Weld L, Goorhuis A, Hamer DH, Schunk M, Jordan S, et al. Travel-related infections presenting in Europe: A 20-year analysis of EuroTravNet surveillance data. Lancet Reg Health Eur. 2021;1:100001.  Gurevitz JM, Antman JG, Laneri K, Morales JM. Temperature, traveling, slums, and housing drive dengue transmission in a non-endemic metropolis. PLoS Negl Trop Dis. 2021;15(6):e0009465.  Hanna JN, Ritchie SA. An apparent recent decline in importations of dengue from Papua New Guinea into north Queensland. Commun Dis Intell Q Rep. 2009;33(1):34-5.  Hanna JN, Richards AR, Esmonde JV, Donohue S, Humphreys JL, Pyke AT, et al. Viraemic importations of dengue into north Queensland, 2009. Commun Dis Intell Q Rep. 2010;34(1):57-8.  Herbinger KH, Drerup L, Alberer M, Nothdurft HD, Sonnenburg F, Loscher T. Spectrum of imported infectious diseases among children and adolescents returning from the tropics and subtropics. J Travel Med. 2012;19(3):150-7.  Hesse EM, Martinez LJ, Jarman RG, Lyons AG, Eckels KH, De La Barrera RA, et al. Dengue Virus Exposures Among Deployed U.S. Military Personnel. Am J Trop Med Hyg. 2017;96(5):1222-6.  Hoffmeister B, Suttorp N, Zoller T. The revised dengue fever classification in German travelers: clinical manifestations and indicators for severe disease. Infection. 2015;43(1):21-8.  Huhtamo E, Korhonen E, Vapalahti O. Imported dengue virus serotype 1 from Madeira to Finland 2012. Euro Surveill. 2013;18(8):21.  Huits R, Angelo KM, Amatya B, Barkati S, Barnett ED, Bottieau E, et al. Clinical Characteristics and Outcomes Among Travelers With Severe Dengue : A GeoSentinel Analysis. Ann Intern Med. 2023;176(7):940-8.  Jones FK, Morrison AM, Santiago GA, Rysava K, Zimler RA, Heberlein LA, et al. Introduction and Spread of Dengue Virus 3, Florida, USA, May 2022-April 2023. Emerg Infect Dis. 2024;30(2):376-9.  Kajimoto Y, Kitajima T. Patient and National Economic Burden of Dengue in Japan: Results from Japanese National Claims Database. Am J Trop Med Hyg. 2020 Jun;102(6):1237-1243.  Khan MA, Rosenberg MG, Fein DM, Quezada XH, Reingold RE, Tadros FK, et al. Internationally Acquired Severe Systemic Infections in Febrile Pediatric Travelers Presenting to the Emergency Department. Pediatr Emerg Care. 2021;37(12):e1315-e20.  Kitro A, Imad HA, Pisutsan P, Matsee W, Sirikul W, Sapbamrer R, et al. Seroprevalence of dengue, Japanese encephalitis and Zika among long-term expatriates in Thailand. J Travel Med. 2024;31(2):01.  Kuan MM, Lin T, Chuang JH, Wu HS. Epidemiological trends and the effect of airport fever screening on prevention of domestic dengue fever outbreaks in Taiwan, 1998-2007. Int J Infect Dis. 2010;14(8):e693-7.  Kutsuna S, Asai Y, Yamamoto K, Shirano M, Konishi K, Asaoka T, et al. Epidemiological trends of imported infectious diseases in Japan: Analysis of imported 2-year infectious disease registry data. J Infect Chemother. 2021;27(4):632-8.  Lagi F, Zammarchi L, Strohmeyer M, Bartalesi F, Mantella A, Meli M, et al. Imported dengue fever in Tuscany, Italy, in the period 2006 to 2012. J Travel Med. 2014;21(5):340-3.  Lau CL, Weinstein P, Slaney D. Dengue surveillance by proxy: travellers as sentinels for outbreaks in the Pacific Islands. Epidemiol Infect. 2013;141(11):2328-34.  Leder K, Torresi J, Brownstein JS, Wilson ME, Keystone JS, Barnett E, et al. Travel-associated illness trends and clusters, 2000-2010. Emerg Infect Dis. 2013;19(7):1049-73.  Li Z, Yin W, Clements A, Williams G, Lai S, Zhou H, et al. Spatiotemporal analysis of indigenous and imported dengue fever cases in Guangdong province, China. BMC Infect Dis. 2012;12:132.  Lindholm DA, Myers T, Widjaja S, Grant EM, Telu K, Lalani T, et al. Mosquito Exposure and Chikungunya and Dengue Infection Among Travelers During the Chikungunya Outbreak in the Americas. Am J Trop Med Hyg. 2017;96(4):903-12.  Liu W, Hu W, Dong Z, You X. Travel-related infection in Guangzhou, China,2009-2019. Travel Med Infect Dis. 2021;43:102106.  Lopez-Amoros AI, Torrus-Tendero D, Merino de Lucas E, Reus Banuls S, Boix Martinez V, Llorens Soriano P, et al. Factors associated with malaria and arboviral disease in patients with imported febrile syndrome: a retrospective cohort study. Emergencias. 2023;35(2):117-24.  Mannestal Johansson C, McBride WJ, Engstrom K, Mills J. Who brings dengue into North Queensland? A descriptive, exploratory study. Aust J Rural Health. 2012;20(3):150-5.  Marks M, Armstrong M, Whitty CJ, Doherty JF. Geographical and temporal trends in imported infections from the tropics requiring inpatient care at the Hospital for Tropical Diseases, London - a 15 year study. Trans R Soc Trop Med Hyg. 2016;110(8):456-63.  Masyeni S, Yohan B, Sasmono RT. Concurrent infections of dengue virus serotypes in Bali, Indonesia. BMC Res Notes. 2019;12(1):129.  Meltzer E, Heyman Z, Bin H, Schwartz E. Capillary leakage in travelers with dengue infection: implications for pathogenesis. Am J Trop Med Hyg. 2012;86(3):536-9.  Mendelson M, Han PV, Vincent P, von Sonnenburg F, Cramer JP, Loutan L, et al. Regional variation in travel-related illness acquired in Africa, March 1997-May 2011. Emerg Infect Dis. 2014;20(4):532-41.  Meynard JB, Ardillon V, Venturin C, Ravachol F, Basurko C, Matheus S, et al. First description of a dengue fever outbreak in the interior of French Guiana, February 2006. Eur J Public Health. 2009;19(2):183-8.  Mizuno Y, Kudo K. Travel-related health problems in Japanese travelers. Travel Med Infect Dis. 2009;7(5):296-300.  Mohammed H, Hayden MH, Lee E, Santiago LM, Krecek RC, Revan F, et al. Dengue in the campus community of an overseas American university: A cross-sectional study. J. 2019;13(3):233-9  Monge-Maillo B, Norman FF, Perez-Molina JA, Navarro M, Diaz-Menendez M, Lopez-Velez R. Travelers visiting friends and relatives (VFR) and imported infectious disease: travelers, immigrants or both? A comparative analysis. Travel Med Infect Dis. 2014;12(1):88-94.  Moore PR, van den Hurk AF, Mackenzie JS, Pyke AT. Dengue viruses in Papua New Guinea: evidence of endemicity and phylogenetic variation, including the evolution of new genetic lineages. Emerg. 2017;6(12):e114.  Norman FF, Henriquez-Camacho C, Diaz-Menendez M, Chamorro S, Pou D, Molina I, et al. Imported Arbovirus Infections in Spain, 2009-2018. Emerg Infect Dis. 2020;26(4):658-66.  Olson D, Birkholz M, Gaensbauer JT, Asturias EJ, Todd JK. Analysis of the pediatric health information system database as a surveillance tool for travel-associated infectious diseases. Am J Trop Med Hyg. 2015;92(5):1067-9.  Onoja AB, Adeniji JA, Olaleye OD. High rate of unrecognized dengue virus infection in parts of the rainforest region of Nigeria. Acta Trop. 2016;160:39-43.  Overbosch FW, Schinkel J, Stolte IG, Prins M, Sonder GJB. Dengue virus infection among long-term travelers from the Netherlands: A prospective study, 2008-2011. PLoS ONE. 2018;13(2):e0192193.  Pandey P, Lee K, Amatya B, Angelo KM, Shlim DR, Murphy H. Health problems in travellers to Nepal visiting CIWEC clinic in Kathmandu - A GeoSentinel analysis. Travel Med Infect Dis. 2021;40:101999.  Parreira R, Conceicao C, Centeno-Lima S, Marques N, Saraiva da Cunha J, Abreu C, et al. Angola's 2013 dengue outbreak: clinical, laboratory and molecular analyses of cases from four Portuguese institutions. J. 2014;8(9):1210-5.  Piyaphanee W, Stoney RJ, Asgeirsson H, Appiah GD, Diaz-Menendez M, Barnett ED, et al. Healthcare seeking during travel: an analysis by the GeoSentinel surveillance network of travel medicine providers. J Travel Med. 2023;30(3):18.  Prince HE, Yeh C, Lape-Nixon M. Primary and probable secondary dengue virus (DV) infection rates in relation to age among DV IgM-positive patients residing in the United States mainland versus the Caribbean islands. Clin Vaccine Immunol. 2012;19(1):105-8.  Rabinowicz S, Schwartz E. Morbidity among Israeli paediatric travellers. J Travel Med. 2017;24(6):01.  Ramadona AL, Tozan Y, Lazuardi L, Rocklov J. A combination of incidence data and mobility proxies from social media predicts the intra-urban spread of dengue in Yogyakarta, Indonesia. PLoS Negl Trop Dis. 2019;13(4):e0007298.  Ratnam I, Black J, Leder K, Biggs BA, Matchett E, Padiglione A, et al. Incidence and seroprevalence of dengue virus infections in Australian travellers to Asia. Eur J Clin Microbiol Infect Dis. 2012;31(6):1203-10.  Redondo-Bravo L, Ruiz-Huerta C, Gomez-Barroso D, Sierra-Moros MJ, Benito A, Herrador Z. Imported dengue in Spain: a nationwide analysis with predictive time series analyses. J Travel Med. 2019;26(8):23.  Ryff KR, Rivera A, Rodriguez DM, Santiago GA, Medina FA, Ellis EM, Torres J, Pobutsky A, Munoz-Jordan J, Paz-Bailey G, Adams LE. Epidemiologic Trends of Dengue in U.S. Territories, 2010-2020. MMWR Surveill Summ. 2023 May 19;72(4):1-12.  Sahni LC, Fischer RSB, Gorchakov R, Berry RM, Payne DC, Murray KO, et al. Arboviral Surveillance among Pediatric Patients with Acute Febrile Illness in Houston, Texas. Am J Trop Med Hyg. 2018;99(2):413-6.  Salmon-Mulanovich G, Blazes DL, Lescano AG, Bausch DG, Montgomery JM, Pan WK. Economic Burden of Dengue Virus Infection at the Household Level Among Residents of Puerto Maldonado, Peru. Am J Trop Med Hyg. 2015;93(4):684-90.  Sanchez-Gonzalez L, Venuto M, Poe S, Major CG, Baskara L, Abdiyeva S, et al. Dengue Virus Infections among Peace Corps Volunteers in Timor-Leste, 2018-2019. Am J Trop Med Hyg. 2021;104(6):2202-9.  Schwartz E, Meltzer E, Mendelson M, Tooke A, Steiner F, Gautret P, et al. Detection on four continents of dengue fever cases related to an ongoing outbreak in Luanda, Angola, March to May 2013. Euro Surveill. 2013;18(21):23.  Schlagenhauf P, Weld L, Goorhuis A, Gautret P, Weber R, von Sonnenburg F, et al. Travel-associated infection presenting in Europe (2008-12): an analysis of EuroTravNet longitudinal, surveillance data, and evaluation of the effect of the pre-travel consultation. Lancet Infect Dis. 2015;15(1):55-64.  Selck FW, Adalja AA, Boddie CR. An estimate of the global health care and lost productivity costs of dengue. Vector Borne Zoonotic Dis. 2014 Nov;14(11):824-6.  Sharp TM, Pillai P, Hunsperger E, Santiago GA, Anderson T, Vap T, et al. A cluster of dengue cases in American missionaries returning from Haiti, 2010. Am J Trop Med Hyg. 2012;86(1):16-22.  Siikamaki HM, Kivela PS, Sipila PN, Kettunen A, Kainulainen MK, Ollgren JP, et al. Fever in travelers returning from malaria-endemic areas: Don't look for malaria only. J Travel Med. 2011;18(4):239-44.  Stidham RA, Cole R, Mabila SL. The four most frequently diagnosed vector-borne diseases among service member and non-service member beneficiaries in the geographic combatant commands, 2010-2022. Msmr. 2024;31(1):14-6.  Stoney RJ, Esposito DH, Kozarsky P, Hamer DH, Grobusch MP, Gkrania-Klotsas E, et al. Infectious diseases acquired by international travellers visiting the USA. J Travel Med. 2018;25(1):01.  Streit JA, Yang M, Cavanaugh JE, Polgreen PM. Upward trend in dengue incidence among hospitalized patients, United States. Emerg Infect Dis. 2011;17(5):914-6.  Sun J, Lu L, Wu H, Yang J, Xu L, Sang S, et al. Epidemiological trends of dengue in mainland China, 2005-2015. Int J Infect Dis. 2017;57:86-91.  Tarantola A, Quatresous I, Ledrans M, Lassel L, Krastinova E, Cordel H, et al. [Imported cases of dengue fever diagnosed in metropolitan France, from January 2001 to December 2006]. Med Mal Infect. 2009;39(1):41-7.  Tilli M, Botta A, Bartoloni A, Corti G, Zammarchi L. Hospitalization for Chagas disease, dengue, filariasis, leishmaniasis, schistosomiasis, strongyloidiasis, and Taenia solium taeniasis/cysticercosis, Italy, 2011-2016. Infection. 2020;48(5):695-713.  Toro C, Trevisi P, Lopez-Quintana B, Amor A, Iglesias N, Subirats M, et al. Imported Dengue Infection in a Spanish Hospital with a High Proportion of Travelers from Africa: A 9-Year Retrospective Study. Am J Trop Med Hyg. 2017;96(3):701-7.  Torres-Fernandez D, Prieto Tato LM, Perez-Ayala A, Moraleda C, Fernandez Cooke E, Blazquez-Gamero D, et al. Etiology and outcome of febrile children coming from the tropics. Enferm Infecc Microbiol Clin (Engl Ed). 2021;39(10):498-502.  Tozan Y, Headley TY, Sewe MO, Schwartz E, Shemesh T, Cramer JP, Eberhardt KA, Ramharter M, Harrison N, Leder K, Angheben A, Hatz C, Neumayr A, Chen LH, De Pijper CA, Grobusch MP, Wilder-Smith A. A Prospective Study on the Impact and Out-of-Pocket Costs of Dengue Illness in International Travelers. Am J Trop Med Hyg. 2019 Jun;100(6):1525-1533.  Tozan Y, Headley TY, Javelle E, Gautret P, Grobusch M, de Pijper C, et al. Impact, healthcare utilization and costs of travel-associated mosquito-borne diseases in international travellers: a prospective study. J Travel Med. 2023;30(7):18.  Trojanek M, Tomickova D, Rohacova H, Kosina P, Gebousky J, Dvorak J, et al. [Dengue fever cases in Czech workers returning from the Maldives]. Epidemiol Mikrobiol Imunol. 2013;62(3):100-5.  Visser JT, Narayanan A, Campbell B. Strongyloides, dengue fever, and tuberculosis conversions in New Zealand police deploying overseas. J Travel Med. 2012;19(3):178-82.  Vlot JA, van Steenbergen JE, Luppino FS, Geary K, van Genderen PJJ, Visser LG. Hospital-based care and/or death followed by repatriation in Dutch travelers: The HAZARD study. Travel Medicine and Infectious Disease. 2022;49.  Wang YL, Wang X, Ren RQ, Zhou L, Tu WW, Ni DX, et al. [Epidemiology of imported infectious diseases in China, 2013-2016]. Chung Hua Liu Hsing Ping Hsueh Tsa Chih. 2017;38(11):1499-503.  Wellington T, Fraser JA, Kuo HC, Hickey PW, Lindholm DA. The Burden of Arboviral Infections in the Military Health System 2012-2019. Am J Trop Med Hyg. 2023;108(5):1007-13.  Wilson ME, Chen LH, Han PV, Keystone JS, Cramer JP, Segurado A, et al. Illness in travelers returned from Brazil: the GeoSentinel experience and implications for the 2014 FIFA World Cup and the 2016 Summer Olympics. Clin Infect Dis. 2014;58(10):1347-56.  Xu M, Chang N, Tu T, Sun J, Jiang J, Xia Y, et al. Economic burden of dengue fever in China: A retrospective research study. PLoS Negl Trop Dis. 2022;16(5):e0010360.  Yang X, Quam MBM, Zhang T, Sang S. Global burden for dengue and the evolving pattern in the past 30 years. J Travel Med. 2021;28(8):29.  Yao MX, Wu SZ, Wang GL, Wang XJ, Fan WJ, Zhang WG, et al. Imported dengue serotype 1 outbreak in a non-endemic region, China, 2017: A molecular and seroepidemiological study. J Infect. 2020;81(2):304-10.  Zammarchi L, Vellere I, Stella L, Bartalesi F, Strohmeyer M, Bartoloni A. Spectrum and burden of neglected tropical diseases observed in an infectious and tropical diseases unit in Florence, Italy (2000-2015). Intern. 2017;12(4):467-77.  Zuckerman NS, Schwartz E, Pandey P, Erster O, Halpern O, Bucris E, et al. Dengue Types 1 and 3 Identified in Travelers Returning from Kathmandu, Nepal, during the October 2022 Outbreak Are Related to Strains Recently Identified in India. Viruses. 2023;15(12):28. |
| **Reason for exclusion: excluded study design (n=14)** |
| Aguiar M, Coelho GE, Rocha F, Mateus L, Pessanha JE, Stollenwerk N. Dengue transmission during the 2014 FIFA World Cup in Brazil. Lancet Infect Dis. 2015;15(7):765-6.  Diaz-Menendez M, Angelo KM, de Miguel Buckley R, Bottieau E, Huits R, Grobusch MP, et al. Dengue outbreak amongst travellers returning from Cuba-GeoSentinel surveillance network, January-September 2022. J Travel Med. 2023;30(2):05.  Eldin C, Ninove L, Drouet H, Gautret P, Leparc-Goffart I, Parola P. Dengue fever type 1 in five travellers returning from the Comoros Islands to Marseille in August 2019 - The risk of importation and subsequent autochthonous dengue transmission in France. Travel Med Infect Dis. 2020;33:101507.  Fabrizio C, Lepore L, Chironna M, Angarano G, Saracino A. Dengue fever in travellers and risk of local spreading: case reports from Southern Italy and literature update. New Microbiol. 2017;40(1):11-8.  Herrero-Martinez JM, Sanchez-Ledesma M, Ramos-Rincon JM. Imported and autochthonous dengue in Spain. Rev Clin Esp (Barc). 2023;223(8):510-9.  Jansen CC, Darbro JM, Birrell FA, Shivas MA, van den Hurk AF. Impact of COVID-19 Mitigation Measures on Mosquito-Borne Diseases in 2020 in Queensland, Australia. Viruses. 2021;13(6):16.  Je S, Bae W, Kim J, Seok SH, Hwang ES. Epidemiological Characteristics and Risk Factors of Dengue Infection in Korean Travelers. J Korean Med Sci. 2016;31(12):1863-73.  Kuna A, Grzybek M. Practical approach to a patient with fever who travelled to the tropics. Pol Arch Intern Med. 2024;134(3):27.  Liebig J, Jansen C, Paini D, Gardner L, Jurdak R. A global model for predicting the arrival of imported dengue infections. PLoS ONE. 2019;14(12):e0225193.  Quam MB, Khan K, Sears J, Hu W, Rocklov J, Wilder-Smith A. Estimating air travel-associated importations of dengue virus into Italy. J Travel Med. 2015;22(3):186-93  Ricco M, Peruzzi S, Balzarini F, Zaniboni A, Ranzieri S. Dengue Fever in Italy: The "Eternal Return" of an Emerging Arboviral Disease. Trop. 2022;7(1):13.  Ritchie SA. Wolbachia and the near cessation of dengue outbreaks in Northern Australia despite continued dengue importations via travellers. J Travel Med. 2018;25(1):01.  Yuan B, Lee H, Nishiura H. Analysis of international traveler mobility patterns in Tokyo to identify geographic foci of dengue fever risk. Theor Biol Med Model. 2021;18(1):17.  Wang B, Liang Y, Yang S, Du Y, Xiong LN, Zhao T, et al. Co-Circulation of 4 Dengue Virus Serotypes among Travelers Entering China from Myanmar, 2017. Emerg Infect Dis. 2018;24(9):1756-8. |
| **Reason for exclusion: no outcome data of interest (n=39)** |
| Aranda C, Martinez MJ, Montalvo T, Eritja R, Navero-Castillejos J, Herreros E, et al. Arbovirus surveillance: first dengue virus detection in local Aedes albopictus mosquitoes in Europe, Catalonia, Spain, 2015. Euro Surveill. 2018;23(47):11.  Aubry M, Teissier Y, Mapotoeke M, Teissier A, Giard M, Musso D, et al. High risk of dengue type 2 outbreak in French Polynesia, 2017. Euro Surveill. 2017;22(14):06.  Baaten GG, Sonder GJ, Zaaijer HL, van Gool T, Kint JA, van den Hoek A. Travel-related dengue virus infection, The Netherlands, 2006-2007. Emerg Infect Dis. 2011;17(5):821-8.  Chu C, Shin ES. Estimation of the Size of Dengue and Zika Infection Among Korean Travelers to Southeast Asia and Latin America, 2016-2017. Osong Public Health Res Perspect. 2019;10(6):394-8.  Fortuna C, Remoli ME, Rizzo C, Benedetti E, Fiorentini C, Bella A, et al. Imported arboviral infections in Italy, July 2014-October 2015: a National Reference Laboratory report. BMC Infect Dis. 2017;17(1):216.  Gautret P, Cramer JP, Field V, Caumes E, Jensenius M, Gkrania-Klotsas E, et al. Infectious diseases among travellers and migrants in Europe, EuroTravNet 2010. Euro Surveill. 2012;17(26):28.  Gobbi F, Capelli G, Angheben A, Giobbia M, Conforto M, Franzetti M, et al. Human and entomological surveillance of West Nile fever, dengue and chikungunya in Veneto Region, Italy, 2010-2012. BMC Infect Dis. 2014;14:60.  Grobusch MP, Weld L, Schnyder JL, Larsen CS, Lindner AK, Popescu CP, et al. COVID-19 impact on EuroTravNet infectious diseases sentinel surveillance in Europe. Travel Med Infect Dis. 2023;53:102583.  Gunaratnam P, Tobin S, Seale H, McAnulty JM. Infectious diseases in returned travellers, NSW, 2010-2011. N S W Public Health Bull. 2014;24(4):171-5.  Herbinger KH, Hanus I, Felbinger TW, Weber C, Beissner M, von Sonnenburg F, et al. Elevated Values of Clinically Relevant Transferases Induced by Imported Infectious Diseases: A Controlled Cross-Sectional Study of 14,559 Diseased German Travelers Returning from the Tropics and Subtropics. Am J Trop Med Hyg. 2016;95(2):481-7.  Herbinger KH, Alberer M, Berens-Riha N, Schunk M, Bretzel G, von Sonnenburg F, et al. Spectrum of Imported Infectious Diseases: A Comparative Prevalence Study of 16,817 German Travelers and 977 Immigrants from the Tropics and Subtropics. Am J Trop Med Hyg. 2016;94(4):757-66.  La Ruche G, Dejour-Salamanca D, Bernillon P, Leparc-Goffart I, Ledrans M, Armengaud A, et al. Capture-recapture method for estimating annual incidence of imported dengue, France, 2007-2010. Emerg Infect Dis. 2013;19(11):1740-8.  Leder K, Mutsch M, Schlagenhauf P, Luxemburger C, Torresi J. Seroepidemiology of dengue in travellers: a paired sera analysis. Travel Med Infect Dis. 2013;11(4):210-3.  Li N, Feng Y, Vrancken B, Chen Y, Dong L, Yang Q, et al. Assessing the impact of COVID-19 border restrictions on dengue transmission in Yunnan Province, China: an observational epidemiological and phylogenetic analysis. Lancet Reg Health West Pac. 2021;14:100259.  Liang H, Luo L, Yang Z, Di B, Bai Z, He P, et al. Re-emergence of dengue virus type 3 in Canton, China, 2009-2010, associated with multiple introductions through different geographical routes. PLoS ONE. 2013;8(2):e55353.  Lim JT, Dickens BL, Ong J, Aik J, Lee VJ, Cook AR, et al. Decreased dengue transmission in migrant worker populations in Singapore attributable to SARS-CoV-2 quarantine measures. J Travel Med. 2021;28(2):23.  Loconsole D, Metallo A, De Robertis AL, Morea A, Quarto M, Chironna M. Seroprevalence of Dengue Virus, West Nile Virus, Chikungunya Virus, and Zika Virus in International Travelers Attending a Travel and Migration Center in 2015-2017, Southern Italy. Vector borne zoonotic dis. 2018;18(6):331-4.  Moncayo AC, Baumblatt J, Thomas D, Harvey KA, Atrubin D, Stanek D, et al. Dengue among American missionaries returning from Jamaica, 2012. Am J Trop Med Hyg. 2015;92(1):69-71.  Moya Notario N, Hernandez-Cabrera M, Carranza-Rodriguez C, Pisos-Alamo E, Jaen-Sanchez N, Perez-Arellano JL. [Febrile syndromes in the traveler returning from tropical regions admitted in a monographic unit]. Rev Esp Quimioter. 2017;30(6):436-42.  Napoli C, Salcuni P, Pompa MG, Declich S, Rizzo C. Estimated imported infections of Chikungunya and Dengue in Italy, 2008 to 2011. J Travel Med. 2012;19(5):294-7.  Nechaev VV, Yarovaya II, Kachenya GV, Doguzhieva EV, Buntovskya SS, Egorickhina AD, et al. Clinical-epidemiological characteristics delivery cases of tropic dengue fever. [Russian]. Jurnal Infektologii. 2021;13(1):78-85.  Nunes MR, Palacios G, Faria NR, Sousa EC, Jr., Pantoja JA, Rodrigues SG, et al. Air travel is associated with intracontinental spread of dengue virus serotypes 1-3 in Brazil. PLoS Negl Trop Dis. 2014;8(4):e2769.  Odolini S, Parola P, Gkrania-Klotsas E, Caumes E, Schlagenhauf P, Lopez-Velez R, et al. Travel-related imported infections in Europe, EuroTravNet 2009. Clin Microbiol Infect. 2012;18(5):468-74.  Overbosch FW, van den Hoek A, Schinkel J, Sonder GJ. High prevalence of previous dengue virus infection among first-generation Surinamese immigrants in the Netherlands. BMC Infect Dis. 2014;14:493.  Polwiang S. The estimation of imported dengue virus from Thailand. J Travel Med. 2015;22(3):194-9.  Riddell A, Babiker ZO. Imported dengue fever in East London: a 6-year retrospective observational study. J Travel Med. 2017;24(3):01.  Rockstroh A, Barzon L, Kumbukgolla W, Su HX, Lizarazo E, Vincenti-Gonzalez MF, et al. Dengue Virus IgM Serotyping by ELISA with Recombinant Mutant Envelope Proteins. Emerg Infect Dis. 2019;25(1):1111-5.  Sanchez-Vegas C, Hamer DH, Chen LH, Wilson ME, Benoit C, Hunsperger E, et al. Prevalence of dengue virus infection in US travelers who have lived in or traveled to dengue-endemic countries. J Travel Med. 2013;20(6):352-60.  Satarvandi D, van der Werff SD, Naucler P, Hildenwall H, Sonden K. Scoring systems for prediction of malaria and dengue fever in non-endemic areas among travellers arriving from tropical and subtropical areas. Emerg Med J. 2024;41(4):242-8.  Semenza JC, Sudre B, Miniota J, Rossi M, Hu W, Kossowsky D, et al. International dispersal of dengue through air travel: importation risk for Europe. PLoS Negl Trop Dis. 2014;8(12):e3278.  Shi Y, Li S, Li X, Zheng K, Yuan S, Huang J. Epidemiological and molecular characterization of dengue viruses imported into Guangzhou during 2009-2013. Springerplus. 2016;5(1):1635.  Shihada S, Emmerich P, Thome-Bolduan C, Jansen S, Gunther S, Frank C, et al. Genetic Diversity and New Lineages of Dengue Virus Serotypes 3 and 4 in Returning Travelers, Germany, 2006-2015. Emerg Infect Dis. 2017;23(2):272-5.  Thai KTD, Wismeijer JA, Van Vugt M, Wolthers KC, De Vries PJ. Dengue fever among ill-returned travellers and concurrent infection by two dengue virus serotypes. Dengue Bulletin. 2009;33(1):60-9.  Valerio L, Roure S, Fernandez-Rivas G, Ballesteros AL, Ruiz J, Moreno N, et al. Arboviral infections diagnosed in a European area colonized by Aedes albopictus (2009-2013, Catalonia, Spain). Travel Med Infect Dis. 2015;13(5):415-21.  van Dodewaard CA, Richards SL. Trends in Dengue Cases Imported into the United States from Pan America 2001-2012. Environ Health Insights. 2015;9:33-40.  Visser JT, Edwards CA. Dengue fever, tuberculosis, human immunodeficiency virus, and hepatitis C virus conversion in a group of long-term development aid workers. J Travel Med. 2013;20(6):361-7.  Warne B, Weld LH, Cramer JP, Field VK, Grobusch MP, Caumes E, et al. Travel-related infection in European travelers, EuroTravNet 2011. J Travel Med. 2014;21(4):248-54.  Watts DM, Rodriguez CM, Palermo PM, Suarez V, Wong SJ, Orbegozo J, et al. Serosurvey for dengue virus infection among pregnant women in the West Nile virus enzootic community of El Paso Texas. PLoS ONE. 2020;15(11):e0242889.  Xu Q, Li ZW, Zhang XA, Liu MY, Wang JL, Zhang HY, et al. The imported infections among foreign travelers in China: an observational study. Global health. 2022;18(1):97. |
| **Hand-searched articles** |
| **Reason for exclusion: excluded population (n=3)** |
| Hadano Y, Shirano M, Goto T. Travel-related illness at a tertiary care hospital in Osaka, Japan. Int J Gen Med. 2016 Oct 20;9:355-359.  Hagmann S, Neugebauer R, Schwartz E, Perret C, Castelli F, Barnett ED, Stauffer WM; GeoSentinel Surveillance Network. Illness in children after international travel: analysis from the GeoSentinel Surveillance Network. Pediatrics. 2010 May;125(5):e1072-80.  Leder K, Torresi J, Libman MD, Cramer JP, Castelli F, Schlagenhauf P, et al. GeoSentinel Surveillance Network. GeoSentinel surveillance of illness in returned travelers, 2007-2011. Ann Intern Med. 2013 Mar 19;158(6):456-68. |
| **Reason for exclusion: no outcome data of interest (n=1)** |
| Naudin J, Blondé R, Alberti C, Angoulvant F, De Lauzanne A, Armoogum P, et al. Aetiology and epidemiology of fever in children presenting to the emergency department of a French paediatric tertiary care centre after international travel. Arch Dis Child. 2012 Feb;97(2):107-11. |
